# First‐trimester screening for pre‐eclampsia and small for gestational age: A comparison of the gaussian and Fetal Medicine Foundation algorithms

**DOI:** 10.1002/ijgo.14306

**Published:** 2022-07-11

**Authors:** Berta Serrano, Erika Bonacina, Carlota Rodo, Pablo Garcia‐Manau, María Ángeles Sanchez‐Duran, María Pancorbo, Cristina Forcada, María Teresa Murcia, Ana Perestelo, Mireia Armengol‐Alsina, Manel Mendoza, Elena Carreras

**Affiliations:** ^1^ Maternal Fetal Medicine Unit, Department of Obstetrics Hospital Universitari Vall d'Hebron, Universitat Autònoma de Barcelona Barcelona Spain

**Keywords:** early‐onset pre‐eclampsia, first trimester, PlGF, pre‐eclampsia, screening, uterine artery doppler

## Abstract

**Objective:**

Pre‐eclampsia (PE) and small for gestational age (SGA) can be predicted from the first trimester. The most widely used algorithm worldwide is the Fetal Medicine Foundation (FMF) algorithm. The recently described Gaussian algorithm has reported excellent results although it is unlikely to be externally validated. Therefore, as an alternative approach, we compared the predictive accuracy for PE and SGA of the Gaussian and FMF algorithms.

**Methods:**

Secondary analysis of a prospective cohort study was conducted at Vall d'Hebron University Hospital (Barcelona) with 2641 singleton pregnancies. The areas under the curve for the predictive performance for early‐onset and preterm PE and early‐onset and preterm SGA were calculated with the Gaussian and FMF algorithms and subsequently compared.

**Results:**

The FMF and Gaussian algorithms showed a similar predictive performance for most outcomes and marker combinations. Nevertheless, significant differences for early‐onset PE prediction favored the Gaussian algorithm in the following combinations: mean arterial blood pressure (MAP) with pregnancy‐associated plasma protein A, MAP with placental growth factor, and MAP alone.

**Conclusions:**

The first‐trimester Gaussian and FMF algorithms have similar performances for PE and SGA prediction when applied with all markers within a routine care setting in a Spanish population, adding evidence to the external validity of the FMF algorithm.

## Synopsis

The first‐trimester Gaussian and Fetal Medicine Foundation algorithms have similar predictive performances for pre‐eclampsia and small‐for‐gestational‐age fetuses.

## 1 INTRODUCTION

Pre‐eclampsia (PE) and small for gestational age (SGA) are the main complications of placental disease. First‐trimester PE screening using algorithms that include a combination of maternal characteristics, biophysical markers (mean arterial blood pressure [MAP] and mean uterine artery pulsatility index [UtAPI]), and biochemical markers (placental growth factor [PlGF] and pregnancy‐associated plasma protein A [PAPP‐A]) can predict PE and SGA.^1^, ^2^, ^3^, ^4^ The Fetal Medicine Foundation (FMF) and Gaussian algorithms can identify 80%–90% of pregnant women who will develop PE with delivery <32/<34 weeks of gestation^1^, ^5^ and 60%–70% of women who will develop PE with delivery <37 weeks,^1^, ^6^ at a 10% false‐positive rate (FPR). These algorithms can also predict 50%–60% of SGA with delivery <32 weeks and 30%–40% of SGA with delivery <37 weeks.^2^, ^4^ Both algorithms use a similar methodology to assess the risk for PE: they combine the a priori risk (based on maternal characteristics and obstetric and medical history) with the results of various biochemical and biophysical markers, to estimate the individual a posteriori risk for PE, which is used to classify a pregnant person as at high or low risk for PE. In both algorithms, risk for PE can be obtained based on maternal factors alone and in combination with any of the biochemical and/or biophysical markers.

Despite the FMF algorithm being the most used and validated worldwide, the Gaussian algorithm has some features that confer advantages in the clinical setting, which is why it has been used for routine first‐trimester PE screening in most maternities in Spain since 2018. First, blood samples for measurements of biochemical markers (PAPP‐A and PlGF) can be drawn between 8 ± 0 weeks and 13 ± 6 weeks, as with routine aneuploidy screening, while in the FMF algorithm biomarkers should be assessed only between 11 ± 0 and 13 ± 6 weeks.^6^ Second, UtAPI assessment can be done both transabdominally and transvaginally, rendering the algorithm more versatile to different clinical settings, as the UtAPI for the FMF algorithm can be assessed only transabdominally. Third, likelihood ratios for the a priori risk calculation were not derived from the study population in which the algorithm was investigated but from a larger meta‐analysis that included >25 million pregnancies.^7^ This may render the Gaussian algorithm less overfitted to a given population and, therefore, more adaptable for populations with different characteristics.

The FMF algorithm has been developed and prospectively validated in large populations, showing comparable predictive performances to the original study.^8^, ^9^, ^10^, ^11^, ^12^ By contrast, the Gaussian algorithm has been investigated only in a single cohort of participants. In the past few years, routine PE screening has been implemented in most hospitals, leaving virtually no women at high risk for PE without aspirin treatment to prospectively assess the external validity of the Gaussian algorithm. Therefore, an indirect approach to test the performance of the Gaussian algorithm is to compare it with the most externally validated combined screening tool for PE worldwide: the FMF algorithm.

The aim of this study was to compare the predictive accuracy for PE and SGA of the Gaussian and FMF algorithms.

## 2 MATERIALS AND METHODS

This is a secondary analysis of previously published data, which was used to test the Gaussian algorithm for early‐onset PE prediction.^3^ That study was approved by the local ethics committee (CEIC‐VHIR PR[AMI]265/2018) and conducted in a prospective fashion at Vall d'Hebron University Hospital (Barcelona) from October 2015 to September 2017.

A total of 3777 unselected singleton pregnant women attending their routine first‐trimester scan (from 11 ± 0 to 13 ± 6 weeks) were invited to participate, and 2946 women agreed and provided their written informed consent. Of those, 305 participants (10.4%) had to be excluded for the following reasons: missing outcome data (*n* = 86), major fetal defects or chromosomopathies (*n* = 13), miscarriage or fetal death <24 weeks (*n* = 15), and insufficient remaining blood sample to measure PlGF (*n* = 191). Before the implementation of the first‐trimester combined screening for PE in 2018, no PE screening was performed at the Vall d'Hebron University Hospital; thus, none of the remaining 2641 participants received aspirin at any time during their pregnancy. Neonatal birthweight was not available for 158 participants; therefore, predictive accuracies for SGA were calculated with 2483 participants and their newborns.

Gestational age was confirmed by fetal crown‐rump length measurement during the first‐trimester scan.^13^ Maternal characteristics and medical and obstetric history were recorded at the first‐trimester ultrasound scan via a patient questionnaire. The following maternal characteristics were recorded: age (years); height (centimeters); weight (kilograms); ethnicity (white European, South American, black, Asian, South‐East Asian, and others); smoking during pregnancy (yes/no); and conception method (spontaneous/assisted reproductive technology/ovulation drugs). Medical history variables included the presence of chronic hypertension (yes/no); diabetes (type 1/type 2/no); renal disease (yes/no); systemic lupus erythematosus (yes/no); and antiphospholipid syndrome (yes/no). Obstetric history variables included parity (nulliparous/multiparous); gestational age at birth (weeks) in the last pregnancy; interval between the last delivery and the beginning of the current one (years); and personal or family history of PE (yes/no). Biochemical markers, including serum PAPP‐A and PlGF, were measured at the first‐trimester routine blood test for aneuploidy screening (from 8 ± 0 to 13 ± 6 weeks) by the fully automated Elecsys assays for PAPP‐A and PlGF on an immunoassay platform (cobas e analyzers, Roche Diagnostics). Biophysical markers, including MAP and UtAPI, were assessed at the first‐trimester scan. Blood pressure was measured automatically using a calibrated device according to a standard procedure: single measurement in one arm (right or left) while women were seated and after a 5‐min rest. MAP was calculated as: diastolic blood pressure + (systolic‐diastolic blood pressure)/3. UtAPI was measured following the recommendations of the FMF.^14^ All examiners were certified by the FMF for PE risk assessment and Doppler ultrasound assessment.

SGA newborns were defined as having a birthweight below the 10th centile according to customized local charts.^15^ Indication for elective delivery was based on Doppler ultrasound findings and conventional cardiotocogram interpretation, according to the current protocol.^16^ Newborns were classified as early SGA if delivery occurred before 32 weeks and as preterm SGA if delivery occurred before 37 weeks.

PE was defined according to the guidelines of the International Society for the Study of Hypertension in Pregnancy: systolic blood pressure ≥ 140 mm Hg and/or diastolic blood pressure ≥ 90 mm Hg, confirmed by repeated measurements over a few hours, developing after 20 weeks in previously normotensive women, accompanied by proteinuria ≥300 mg in 24 h, spot urine protein/creatinine ratio ≥0.3 mg/mg, or dipstick urinalysis ≥1+ when a quantitative method was not available.^17^ Early‐onset and preterm PE were defined as PE requiring delivery before 34 and 37 weeks, respectively.

For the Gaussian algorithm, multiples of the median (MoMs) for each marker were calculated according to the methodology described in a previous study.^3^ For the FMF algorithm, MoMs were obtained using the batch calculation tool provided in the FMF website.^18^ We then coded the variables required for the prediction formulas according to the description provided in the corresponding published articles.^1^, ^3^ For the Gaussian algorithm, the prenatal screening software SsdwLab 6 (SBP Soft 2007 S.L) was used to calculate early‐onset PE probability scores. For the FMF algorithm, the risk calculation tool provided in the FMF website was used.^19^

Besides the a priori risks, the four markers (PAPP‐A, PlGF, MAP, and UtAPI) can be incorporated alone or in combination of two, three, or four for risk calculation, depending on the markers available in the clinical practice. Therefore, there are 15 possible marker combinations. Nevertheless, only the seven most clinically relevant have been investigated in this study (MAP alone, MAP + PlGF, MAP + UtAPI, MAP + PAPP‐A, MAP + UtAPI + PAPP‐A, MAP + UtAPI + PlGF, and MAP + UtAPI + PlGF + PAPP‐A).

### 2.1 Statistical Analysis

The statistical software RStudio Team (version 1.2.5033 [2019], RStudio: Integrated Development for R. RStudio, Inc.) was used for statistical analysis. Categorical data were reported as frequency and percentage, and comparisons between groups were performed by chi‐square or Fisher tests, as appropriate. Continuous variables were reported as the median and interquartile range, and the Mann–Whitney *U* test was used to assess differences between groups. Receiver operating characteristic (ROC) curves were generated and detection rates (DRs) at fixed 5%, 10%, 15%, 20%, 25%, and 30% FPRs were calculated for both algorithms. The predictive accuracies of both algorithms were compared for a fixed FPR of 10% as well as for the resulting areas under the curve (AUC), which were compared by the Delong test.^20^ Bonferroni correction was used in all tests when multiple comparisons were assessed. Statistical significance was set at *P* < 0.05.

## 3 RESULTS

Among the 2641 participants, 30 (1.14%) women developed preterm PE, including 11 (0.42%) with early‐onset PE. Among the 2483 newborns, 44 (1.77%) were preterm SGA, including 8 (0.32%) with early‐onset SGA.

Characteristics of the study population are summarized in Table 1 and Table 2.

**TABLE 1:** Baseline characteristics of the study population based on PE outcome

	PE < 34 weeks	PE < 37 weeks	No PE < 37 weeks
	(*n* = 11)	(*n* = 30)	(*n* = 2611)
Age (years)	34 (32–37)	35.5 (31–38)^a^	32 (28–36)^c^
BMI	23.1 (22.5–32.1)	24.0 (22.5–27.6)	23.8 (21.3–27.5)
Ethnicity			
White	10 (90.9%)	25 (83.3%)	2209 (84.6%)
Black	0 (0.0%)	1 (3.3%)	71 (2.72%)
Mixed	1 (9.1%)	2 (6.7%)	209 (8.0%)
Asian	0 (0.0%)	2 (6.7%)	63 (2.41%)
Southeast Asian	0 (0.0%)	0 (0.0%)	59 (2.26%)
Smoking during pregnancy	1 (9.1%)	3 (10.0%)	309 (11.8%)
ART	1 (9.1%)	2 (6.7%)	93 (3.6%)
Insemination	1 (9.1%)	1 (3.3%)	16 (0.6%)
IVF	0 (0.0%)	1 (3.3%)	77(2.95%)
IVF with egg donation	0 (0.0%)	1 (3.3%)	25 (0.96%)
Medical history			
Chronic hypertension	3 (27.3%)^a^	5 (16.7%)^a^	24 (0.9%)^b^ ^,^ ^c^
Diabetes	0 (0.0%)	1 (3.3%)	35 (1.3%)
Autoimmune disease	0 (0.0%)	3 (10.0%)	105 (4.0%)
APS	0 (0.0%)	1 (3.3%)	8 (0.3%)
Obstetric history			
Nulliparous	2 (18.2%)	13 (43.3%)	1219 (46.7%)
Previous PE	2 (18.2%)^a^	5 (16.7%)^a^	30 (1.1%)^b^ ^,^ ^c^
Biophysical variables			
GA at the time of first‐trimester ultrasound scan (weeks)	12.7 (12.3–13.3)	12.7 (12.3–13.3)	12.6 (12.1–13)
MAP (mm Hg)	96 (88.3–104.3)^a^	91.2 (86.7–97.3)^a^	84.3 (78.7–90.7)^b^ ^,^ ^c^
MoM MAP	1.14 (1.10–1.37)^a^	1.14 (1.10–1.29)^a^	1.06 (0.97–1.14)^b^ ^,^ ^c^
Mean UtAPI	2.25 (1.89–3.05)^a^ ^,^ ^c^	1.91 (1.71–2.31)^a^ ^,^ ^b^	1.68 (1.34–2.05)^b^ ^,^ ^c^
MoM UtAPI	1.32 (1.12–2.13)^a^	1.19 (1.01–1.44)^a^	1.03 (0.84–1‐26)^b^ ^,^ ^c^
Biochemical variables			
GA for PAPP‐A + PlGF measurement	11.4 (9.9–12.3)	10.9 (9.9–11.7)	10.6 (10–11.3)
PAPP‐A (mU/L)	1373 (607.3–2291)	1158 (602.3–2291)	1358 (823.2–2370)
MoM PAPP‐A	0.73 (0.6–0.93)^a^	0.72 (0.57–1.05)^a^	1.05 (0.73–1.5)^b^ ^,^ ^c^
PlGF (pg/ml)	22.3 (19.0–29.8)^a^	25.0 (19.3–31.7)^a^	32.2 (24.3–43.0)^b^ ^,^ ^c^
MoM PlGF	0.69 (0.52–1.05)^a^	0.78 (0.63–0.98)^a^	0.96 (0.76–1.19)^b^ ^,^ ^c^

*Note*: Categorical data are reported as frequency (percentage) and continuous data as median (interquartile range).

Abbreviations: APS, antiphospholipid syndrome; ART, assisted reproductive technique; BMI, body mass index; GA, gestational age; IVF, in vitro fertilization; MAP, mean arterial pressure; MoM, multiple of median; PAAP‐A, pregnancy‐associated plasma protein A; PlGF, placental growth factor; UtAPI, uterine artery pulsatility index.

^a^ Significant difference as compared with unaffected women.

^b^ Significant difference as compared with early‐onset pre‐eclampsia (PE).

^c^ Significant difference as compared with women with preterm PE.

**TABLE 2:** Baseline characteristics of the study population based on SGA outcome

	SGA < 32 weeks	SGA < 37 weeks	No SGA < 37 weeks
	(*n* = 8)	(*n* = 44)	(*n* = 2439)
Age (years)	31.5 (29–33)	32 (28.5–37)	32 (28–36)
BMI	23.1 (21.9–24.5)	23.1 (20.2–26.4)	23.9 (21.4–27.6)
Ethnicity			
White	159 (94.6%)	189 (93.6%)	2196 (84.6%)
Black	4 (2.4%)	6 (3.0%)	70 (2.7%)
Mixed	5 (3.0%)	5 (2.5%)	209 (8.1%)
Asian	0 (0.0%)	1 (0.5%)	64 (2.5%)
Southeast Asian	0 (0.0%)	1 (0.5%)	58 (2.2%)
Smoking during pregnancy	0 (0.0%)	15 (34.1%)^a^	283 (11.6%)^c^
ART			
Insemination	2 (1.2%)	3 (1.5%)	16 (0.6%)
IVF	6 (3.6%)	7 (3.5%)	77 (3.0%)
IVF with egg donation	0 (0.0%)	1 (2.3%) (% del total)	22 (0.9%)
Medical history			
Chronic hypertension	1 (12.5%)^a^	2 (4.5%)	27 (1.1%)^b^
Diabetes	0 (0.0%)	2 (4.5%)	32 (1.3%)
Autoimmune disease	1 (12.5%)	2 (4.5%)	101 (4.1%)
APS	0 (0.0%)^a^	3 (6.8%)	6 (0.2%)^b^
Obstetric history			
Nulliparous	5 (62.5%)	20 (45.5%)	1126 (46.2%)
Previous PE	1 (12.5%)^a^	2 (4.5%)	32 (1.3%)^b^
Biophysical variables			
GA at the time of first‐trimester ultrasound scan (weeks)	12.4 (12.1–12.6)	12.4 (11.9–12.9)	12.6 (12.1–13)
MAP (mm Hg)	90.8 (85.2–96)	86.7 (80–91.1)	84.3 (78.3–90.7)
MoM MAP	1.14 (1.04–1.17)	1.07 (0.96–1.17)	1.05 (0.97–1.15)
Mean UtAPI	1.88 (1.74–2.67)	1.94 (1.72–2.45)^a^	1.68 (1.34–2.04)^c^
MoM UtAPI	1.12 (1.01–1.60)	1.20 (1.02–1.47)^a^	1.02 (0.84–1.25)^c^
Biochemical variables			
GA for PAPP‐A + PlGF measurement	11.4 (10.4–12.3)	10.7 (10–11.8)	10.6 (10–11.3)
PAPP‐A (mU/L)	1801 (932.2–2456)	964.25 (631.0–1794.5)	1355 (816–2387)
MoM PAPP‐A	0.74 (0.6–0.89)	0.73 (0.55–1.1)^a^	1.06 (0.73–1.51)^c^
PlGF (pg/ml)	20.0 (18.1–26.1)^a^	28.2 (19.5–38.4)	32.1 (24.1–43.0)^b^
MoM PlGF	0.60 (0.42–0.79)^a^	0.72 (0.61–0.97)^a^	0.96 (0.75–1.18)^b^ ^,^ ^c^

*Note*: Categorical data are reported as frequency (percentage) and continuous data as median (interquartile range).

Abbreviations: APS, antiphospholipid syndrome; ART, assisted reproductive technique; BMI, body mass index; GA, gestational age; IVF, in vitro fertilization; MAP, mean arterial pressure; MoM, multiple of median; PAAP‐A, pregnancy‐associated plasma protein A; PE, pre‐eclampsia; PlGF, placental growth factor; UtAPI, uterine artery pulsatility index.

^a^ Significant difference as compared with unaffected women.

^b^ Significant difference as compared with early‐onset small for gestational age (SGA).

^c^ Significant difference compared with preterm SGA.

For prediction of early‐onset and preterm PE, and early‐onset and preterm SGA, the Gaussian and FMF algorithms showed a similar predictive performance with all marker combinations, except for early‐onset PE prediction with MAP and PAPP‐A (Gaussian AUC = 0.833 [95% CI, 0.727–0.939] vs FMF AUC = 0.771 [95% CI, 0.631–0.911]; *P* = 0.002), MAP and PlGF (Gaussian AUC = 0.905 [95% CI, 0.844–0.965] vs FMF AUC = 0.858 [95% CI, 0.768–0.947]; *P* = 0.01), and MAP alone (Gaussian AUC = 0.795 [95% CI, 0.679–0.912] vs FMF AUC = 0.758 [95% CI, 0.621–0.895]; *P* = 0.02), where the FMF algorithm showed a significantly lower AUC Tables 3, 4, 5, 6.

**TABLE 3:** DR and AUC for prediction of early‐onset PE by the Gaussian and the FMF algorithms

A priori risk +	PE < 34 ± 0 weeks (n = 11)														*P* value
	Gaussian algorithm							FMF algorithm							
	AUC (95% CI)	DR at 5% FPR (95% CI)	DR at 10% FPR (95% CI)	DR at 15% FPR (95% CI)	DR at 20% FPR (95% CI)	DR at 25% FPR (95% CI)	DR at 30% FPR (95% CI)	AUC (95% CI)	DR at 5% FPR (95% CI)	DR at 10% FPR (95% CI)	DR at 15% FPR (95% CI)	DR at 20% FPR (95% CI)	DR at 25% FPR (95% CI)	DR at 30% FPR (95% CI)	
MAP	0.795 (0.679–0.912)	36.4 (9.09–63.6)	45.5 (18.2–72.7)	54.6 (27.3–81.8)	54.6 (27.3–81.8)	72.7 (45.5–100.0)	72.7 (45.5–100.0)	0.758 (0.621–0.895)	27.3 (0.0–54.6)	27.3 (0.0–54.6)	27.3 (9.09–63.6)	54.6 (27.3–90.9)	72.7 (45.5–100.0)	72.7 (45.5–100.0)	0.0214
MAP + PlGF	0.905 (0.844–0.965)	36.4 (9.09–63.6)	63.6 (36.4–90.9)	81.8 (54.6–100.0)	81.8 (54.6–100.0)	81.8 (54.6–100.0)	90.9 (72.7–100.0)	0.858 (0.768–0.947)	45.5 (18.2–72.7)	45.5 (18.2–72.7)	63.6 (36.4–90.9)	72.7 (45.5–100.0)	81.8 (54.6–100.0)	81.8 (54.6–100.0)	0.0112
MAP + UtAPI	0.908 (0.840–0.975)	63.6 (36.4–90.9)	63.6 (36.4–90.9)	63.6 (36.4–90.9)	72.7 (45.5–100.0)	90.9 (72.7–100.0)	100.0 (100.0–100.0)	0.868 (0.775–0.961)	45.5 (18.2–72.7)	54.6 (27.3–81.8)	63.6 (36.4–90.9)	72.7 (45.5–100.0)	81.8 (54.6–100.0)	81.8 (54.6–100.0)	0.1059
MAP + PAPP‐A	0.833 (0.727–0.939)	36.4 (9.09–63.6)	54.6 (27.3–81.8)	54.6 (27.3–81.8)	72.7 (45.5–95.6)	72.7 (45.5–95.6)	72.7 (45.5–95.6)	0.771 (0.631–0.911)	27.3 (0.0–54.6)	27.3 (0.0–54.6)	54.6 (27.3–81.8)	63.6 (36.4–90.9)	72.7 (45.5–95.6)	72.7 (45.5–95.6)	0.0022
MAP + UtAPI + PAPP‐A	0.910 (0.844–0.977)	63.6 (36.4–90.9)	63.6 (36.4–90.9)	72.7 (45.5–100.0)	72.7 (45.5–100.0)	81.8 (54.6–100.0)	100.0 (100.0–100.0)	0.870 (0.768–0.972)	45.5 (18.2–72.3)	54.6 (27.3–81.8)	72.7 (45.5–90.9)	81.8 (54.6–100.0)	81.8 (54.6–100.0)	81.8 (54.6–100.0)	0.1374
MAP + UtAPI + PlGF	0.951 (0.919–0.983)	54.6 (27.3–81.8)	81.8 (54.5–100.0)	90.9 (72.7–100.0)	100.0 (100.0–100.0)	100.0 (100.0–100.0)	100.0 (100.0–100.0)	0.923 (0.864–0.982)	63.6 (36.4–90.9)	72.7 (45.5–100.0)	72.7 (45.5–100.0)	90.9 (72.7–100.0)	90.9 (72.7–100.0)	90.9 (72.7–100.0)	0.1325
MAP + UtAPI + PlGF + PAPP‐A	0.945 (0.912–0.979)	54.6 (27.3–81.8)	81.8 (54.6–100.0)	90.9 (72.7–100.0)	100.0 (100.0–100.0)	100.0 (100.0–100.0)	100.0 (100.0–100.0)	0.945 (0.908–0.982)	54.6 (27.3–81.8)	90.9 (54.6–100.0)	90.9 (72.7–100.0)	90.9 (72.7–100.0)	100 (100–100)	100 (100–100)	0.9651

*Note*: Comparisons between areas under the curve (AUCs) were performed by two‐tailed *P* values.

Abbreviations: CI, confidence interval; DR, detection rate; FMF, Fetal Medicine Foundation; FPR, false‐positive rate; MAP, mean arterial pressure; PAPP‐A, pregnancy‐associated plasma protein A; PE, pre‐eclampsia; PlGF, placental growth factor; UtAPI, mean uterine artery pulsatility index.

**TABLE 4:** DR and AUC for prediction of preterm PE by the Gaussian and the FMF algorithms

A priori risk +	PE < 37 ± 0 weeks (*n* = 30)														*P* value
	Gaussian algorithm							FMF algorithm							
	AUC (95% CI)	DR at 5% FPR (95% CI)	DR at 10% FPR (95% CI)	DR at 15% FPR (95% CI)	DR at 20% FPR (95% CI)	DR at 25% FPR (95% CI)	DR at 30% FPR (95% CI)	AUC (95% CI)	DR at 5% FPR (95% CI)	DR at 10% FPR (95% CI)	DR at 15% FPR (95% CI)	DR at 20% FPR (95% CI)	DR at 25% FPR (95% CI)	DR at 30% FPR (95% CI)	
MAP	0.737 (0.648–0.827)	0.2667 (13.3–43.3)	36.7 (20.0–53.3)	50.0 (33.3–66.7)	53.3 (33.3–70.0)	56.7 (40.0–73.3)	63.3 (46.7–80.0)	0.727 (0.637–0.817)	26.7 (10.0–43.3)	26.7 (13.3–46.7)	36.7 (20.0–53.3)	50.0 (33.3–70.0)	60.0 (43.3–76.7)	60.0 (43.3–80.0)	0.3884
MAP + PlGF	0.802 (0.722–0.881)	26.7 (13.3–43.3)	46.7 (30.0–63.3)	60.0 (43.3–76.6)	66.7 (50.0–82.2)	73.3 (53.3–86.7)	76.7 (60.0–90.0)	0.790 (0.712–0.868)	36.7 (20.0–53.3)	40.0 (23.3–60.0)	53.3 (33.3–70.0)	60.0 (43.3–76.7)	66.7 (50.0–83.3)	66.7 (50.0–83.3)	0.4292
MAP + UtAPI	0.782 (0.692–0.872)	36.7 (20.0–53.3)	40.0 (23.3–56.7)	46.7 (30.0–63.3)	56.7 (36.7–76.7)	76.7 (60.0–90.0)	80.0 (63.3–93.3)	0.786 (0.701–0.871)	30.0 (13.3–50.0)	43.3 (26.7–63.3)	46.7 (30.0–63.3)	63.3 (46.7–80.0)	70.0 (53.3–86.7)	73.3 (56.7–86.7)	0.8590
MAP + PAPP‐A	0.773 (0.687–0.859)	33.3 (20.0–50.0)	43.3 (26.7–63.3)	53.3 (33.3–70.0)	63.3 (46.7–80.0)	63.3 (46.7–80.0)	63.3 (46.7–80.0)	0.747 (0.658–0.836)	23.3 (10.0–40.0)	36.7 (20.0–53.3)	50.0 (33.3–66.7)	53.3 (36.7–70.0)	60.0 (40.0–76.7)	63.3 (43.3–80.0)	0.0955
MAP + UtAPI + PAPP‐A	0.797 (0.708–0.886)	36.7 (20.0–53.3)	43.3 (26.7–60.0)	53.3 (36.7–73.3)	70.0 (53.3–83.4)	76.7 (56.7–90.0)	83.3 (70.0–96.7)	0.800 (0.714–0.887)	36.7 (16.7–53.3)	50.0 (33.3–66.7)	56.7 (40.0–73.3)	70.0 (50.0–86.7)	76.7 (60.0–90.0)	76.7 (60.0–90.0)	0.8846
MAP + UtAPI + PlGF	0.798 (0.704–0.893)	36.7 (20.0–56.7)	46.7 (30.0–66.7)	56.7 (40.0–73.3)	80.0 (63.3–93.3)	80.0 (63.3–93.3)	80.0 (63.3–93.3)	0.818 (0.739–0.897)	36.7 (20.0–53.3)	50.0 (30.0–66.7)	56.7 (40.0–73.3)	66.7 (50.0–83.3)	80.0 (63.3–93.3)	80.0 (66.7–93.3)	0.4780
MAP + UtAPI + PlGF + PAPP‐A	0.782 (0.683–0.882)	33.3 (16.7–50.0)	46.7 (30.0–66.7)	63.3 (46.7–80.0)	76.7 (60.0–90.0)	76.7 (60.0–90.0)	76.7 (60.0–90.0)	0.818 (0.728–0.907)	36.7 (20.0–56.7)	63.4 (43.3–80.0)	70.0 (53.3–86.7)	73.3 (53.3–86.7)	76.7 (60.0–90.0)	76.7 (60.0–93.3)	0.3467

*Note*: Comparisons between areas under the curve (AUCs) were performed by two‐tailed *P* values.

Abbreviations: CI, confidence interval; DR, detection rate; FMF, Fetal Medicine Foundation; FPR, false‐positive rate; MAP, mean arterial pressure; PAPP‐A, pregnancy‐associated plasma protein A; PE, pre‐eclampsia; PlGF, placental growth factor; UtAPI, mean uterine artery pulsatility index.

**TABLE 5:** DR and AUC for prediction of early‐onset SGA by the Gaussian and the FMF algorithms

A priori risk +	SGA < 32 ± 0 weeks (*n* = 8)														*P* value
	Gaussian algorithm							FMF algorithm							
	AUC (95% CI)	DR at 5% FPR (95% CI)	DR at 10% FPR (95% CI)	DR at 15% FPR (95% CI)	DR at 20% FPR (95% CI)	DR at 25% FPR (95% CI)	DR at 30% FPR (95% CI)	AUC (95% CI)	DR at 5% FPR (95% CI)	DR at 10% FPR (95% CI)	DR at 15% FPR (95% CI)	DR at 20% FPR (95% CI)	DR at 25% FPR (95% CI)	DR at 30% FPR (95% CI)	
MAP	0.700 (0.546–0.854)	12.5 (0.0–37.5)	12.5 (0.0–37.5)	37.5 (0.0–75.0)	37.5 (12.5–75.0)	62.5 (25.0–87.8)	62.5 (25.0–87.8)	0.722 (0.604–0.841)	12.5 (0.0–37.5)	12.5 (0.0–37.5)	12.5 (0.0–37.5)	37.5 (12.5–75.0)	62.5 (25.0–87.8)	62.5 (25.0–87.8)	0.4854
MAP + PlGF	0.840 (0.710–0.970)	25.0 (0.0–62.5)	37.5 (12.5–75.0)	75.0 (37.5–100.0)	75.0 (37.5–100.0)	87.5 (62.5–100)	87.5 (62.5–100)	0.865 (0.784–0.945)	37.5 (0.0–75.0)	37.5 (12.5–75.0)	50.0 (12.5–87.5)	87.5 (50.0–100.0)	87.5 (62.5–100)	87.5 (62.5–100)	0.4625
MAP + UtAPI	0.740 (0.564–0.916)	25.0 (0.0–62.5)	37.5 (12.5–75.0)	37.5 (12.5–75.0)	50.0 (12.5–87.5)	62.5 (25.0–87.8)	62.5 (25.0–100)	0.777 (0.655–0.898)	25.0 (0.0–62.5)	25.0 (0.0–62.5)	37.5 (12.5–75.0)	50.0 (12.5–87.5)	62.5 (25.0–87.8)	62.5 (25.0–87.8)	0.4147
MAP + PAPP‐A	0.743 (0.581–0.905)	12.5 (0.0–37.5)	37.5 (1.6–75.0)	37.5 (12.5–75.0)	62.5 (25.0–87.8)	62.5 (25.0–100)	62.5 (25.0–100)	0.746 (0.619–0.873)	12.5 (0.0–37.5)	12.5 (0.0–37.5)	37.5 (12.2–75.0)	50.0 (12.5–87.5)	62.5 (25.0–87.8)	62.5 (25.0–87.8)	0.9418
MAP + UtAPI + PAPP‐A	0.757 (0.589–0.925)	25.0 (0.0–62.5)	37.5 (0.0–75.0)	50.0 (12.5–87.5)	50.0 (12.5–87.5)	50.0 (12.5–87.5)	75.0 (49.7–100.0)	0.795 (0.663–0.926)	25.0 (0.0–62.5)	37.5 (0.0–75.5)	50.0 (12.5–87.5)	62.5 (25.0–87.5)	62.5 (25.0–87.5)	75.0 (37.5–100)	0.4514
MAP + UtAPI + PlGF	0.811 (0.641–0.982)	37.5 (12.5–75.0)	62.5 (25.5–87.5)	75.0 (50.0–100.0)	75.0 (50.0–100.0)	75.0 (50.0–100.0)	75.0 (50.0–100.0)	0.875 (0.774–0.976)	62.5 (25.0–87.5)	62.5 (25.0–87.5)	62.5 (25.0–87.5)	75.0 (37.5–100.0)	75.0 (37.5–100.0)	87.5 (50.0–100.0)	0.1289
MAP + UtAPI + PlGF + PAPP‐A	0.806 (0.635–0.978)	37.5 (12.5–75.0)	62.5 (25.5–87.5)	75.0 (37.5–100.0)	75.0 (37.5–100.0)	75.0 (37.5–100.0)	75.0 (37.5–100.0)	0.906 (0.834–0.978)	50.0 (12.5–87.5)	75.0 (37.5–100.0)	75.0 (50.0–100.0)	75.0 (50.0–100.0)	87.5 (62.5–100.0)	100 (100–100)	0.0582

*Note*: Comparisons between areas under the curve (AUCs) were performed by two‐tailed *P* values.

Abbreviations: CI, confidence interval; DR, detection rate; FMF, Fetal Medicine Foundation; FPR, false‐positive rate; MAP, mean arterial pressure; PAPP‐A, pregnancy‐associated plasma protein A; PlGF, placental growth factor; SGA, small for gestational age; UtAPI, mean uterine artery pulsatility index.

**TABLE 6:** DR and AUC for prediction of preterm SGA by the Gaussian and the FMF algorithms

A priori risk +	SGA < 37 ± 0 weeks (*n* = 44)														*P* value
	Gaussian algorithm							FMF algorithm							
	AUC (95% CI)	DR at 5% FPR (95% CI)	DR at 10% FPR (95% CI)	DR at 15% FPR (95% CI)	DR at 20% FPR (95% CI)	DR at 25% FPR (95% CI)	DR at 30% FPR (95% CI)	AUC (95% CI)	DR at 5% FPR (95% CI)	DR at 10% FPR (95% CI)	DR at 15% FPR (95% CI)	DR at 20% FPR (95% CI)	DR at 25% FPR (95% CI)	DR at 30% FPR (95% CI)	
MAP	0.546 (0.459–0.632)	9.1 (0.7–18.2)	18.2 (6.8–29.6)	22.7 (11.4–36.4)	22.7 (11.4–36.4)	29.6 (15.9–43.2)	36.4 (22.7–50.0)	0.563 (0.477–0.649)	9.1 (2.3–18.2)	13.6 (4.5–25.0)	18.2 (9.1–29.6)	27.3 (15.9–40.9)	34.1 (20.5–47.7)	36.4 (22.7–52.3)	0.4230
MAP + PlGF	0.630 (0.540–0.719)	9.1 (2.3–20.5)	20.5 (9.1–31.9)	38.2 (22.7–52.3)	43.2 (29.6–56.8)	45.5 (31.8–61.4)	52.3 (38.6–65.9)	0.651 (0.562–0.739)	13.6 (4.5–25.0)	22.7 (11.4–36.4)	36.4 (22.7–52.3)	43.2 (29.6–59.1)	50.0 (36.4–65.9)	54.6 (38.6–68.2)	0.3766
MAP + UtAPI	0.653 (0.57–0.737)	15.9 (6.8–27.3)	25.0 (13.6–36.7)	29.6 (18.2–45.5)	36.4 (22.7–52.3)	52.3 (36.4–65.9)	54.6 (40.9–70.5)	0.634 (0.547–0.722)	13.6 (4.5–25.0)	20.5 (9.1–34.1)	27.3 (15.9–43.2)	38.7 (25.0–54.6)	45.5 (31.8–59.2)	50.0 (36.4–65.9)	0.4437
MAP + PAPP‐A	0.592 (0.505–0.678)	7.9 (2.3–18.2)	22.7 (11.4–34.1)	25.0 (13.6–38.6)	34.1 (22.5–50.0)	38.6 (25.0–52.3)	40.9 (27.3–56.8)	0.591 (0.504–0.677)	6.8 (0.0–15.9)	11.4 (4.5–22.7)	27.3 (13.6–40.9)	34.1 (20.5–47.7)	36.4 (22.7–50.0)	45.5 (29.6–61.4)	0.9680
MAP + UtAPI + PAPP‐A	0.670 (0.587–0.752)	15.9 (6.8–27.3)	20.5 (9.1–34.1)	29.5 (15.9–43.2)	43.2 (29.6–61.4)	52.3 (36.4–65.9)	61.4 (47.7–75.0)	0.661 (0.575–0.746)	13.6 (4.3–25.0)	25.0 (11.4–38.6)	34.1 (20.5–47.7)	40.9 (25.0–56.8)	47.7 (34.1–63.4)	56.8 (40.9–70.5)	0.7167
MAP + UtAPI + PlGF	0.697 (0.612–0.782)	20.5 (9.1–34.0)	29.5 (15.9–43.2)	45.5 (31.8–59.1)	54.6 (38.6–68.2)	59.1 (43.2–72.7)	63.6 (47.7–77.3)	0.689 (0.601–0.776)	18.2 (6.8–31.8)	34.1 (20.5–50.0)	45.5 (31.8–59.2)	47.8 (34.1–63.6)	56.8 (40.9–70.5)	59.1 (43.2–72.7)	0.7524
MAP + UtAPI + PlGF + PAPP‐A	0.684 (0.598–0.769)	18.2 (6.8–29.6)	29.5 (15.9–43.2)	43.2 (27.3–59.1)	52.3 (36.4–65.9)	54.6 (38.6–70.5)	61.4 (47.7–75.0)	0.727 (0.645–0.809)	22.7 (11.4–38.6)	40.9 (25.0–56.8)	47.7 (34.1–63.6)	52.3 (36.4–65.9)	63.6 (49.9–79.5)	68.2 (54.6–81.8)	0.1749

*Note*: Comparisons between areas under the curve (AUCs) were performed by two‐tailed *P* values.

Abbreviations: CI, confidence interval; DR, detection rate; FMF, Fetal Medicine Foundation; FPR, false‐positive rate; MAP, mean arterial pressure; PAPP‐A, pregnancy‐associated plasma protein A; PlGF, placental growth factor; SGA, small for gestational age; UtAPI, mean uterine artery pulsatility index.

For early‐onset PE prediction, the Gaussian algorithm showed the greatest AUC when combining maternal history, MAP, UtAPI and PlGF (0.951; 95% CI, 0.919–0.983), followed by the combination of all markers (0.945; 95% CI, 0.912–0.979). The FMF algorithm showed the greatest AUC when combining all markers (0.945; 95% CI, 0.908–0.982).

For preterm PE prediction, the Gaussian algorithm showed the greatest AUC when combining maternal history, MAP and PlGF (0.802; 95% CI, 0.722–0.881), followed by the combination of all markers without PAPP‐A (0.798; 95% CI, 0.704–0.893). The FMF algorithm showed the greatest AUC when combining all markers (0.818; 95% CI, 0.728–0.907).

For early‐onset SGA prediction, the Gaussian algorithm showed the greatest AUC when combining maternal history, MAP and PlGF (0.840; 95% CI, 0.710–0.970), followed by the combination of all markers without PAPP‐A (0.811; 95% CI, 0.641–0.982). The FMF algorithm showed the greatest AUC when combining all markers (0.906; 95% CI, 0.834–0.978).

For preterm SGA prediction, the Gaussian algorithm showed the greatest AUC when combining maternal history, MAP, UtAPI, and PlGF (0.697; 95% CI, 0.612–0.782), followed by the combination of all markers (0.684; 95% CI, 0.598–0.769). The FMF algorithm showed the greatest AUC when combining all markers (0.727; 95% CI, 0.645–0.809).

## 4 DISCUSSION

This study shows that the Gaussian and FMF algorithms have similar predictive accuracies for PE and SGA, except for early‐onset PE, where the FMF algorithm showed a significantly lower AUC with the combinations of MAP and PAPP‐A, MAP and PlGF, and MAP alone. These significant differences could be partly attributed to the different methodology required for MAP assessment in both algorithms. In this study, MAP was measured once in only one arm and after a 5‐min rest, while the FMF algorithm was designed with an average of two MAP measurements performed at 1‐min intervals in both arms simultaneously after a 5‐min rest.^21^ This different methodology for MAP measurements may have affected the accuracy of all combinations including MAP in the FMF algorithm, but especially MAP alone or those combinations that included MAP with one other factor.

The FMF algorithm has been externally validated by several studies in various populations, showing comparable performance to that of the original study. Nevertheless, one study showed that some algorithms could underperform when applied to populations that were different to the population where they were developed.^22^ In this study, we show that performance of the FMF algorithm in a Spanish population was similar to the performance obtained in the original study, further supporting the external validity of the FMF algorithm. By contrast, the predictive ability of the Gaussian algorithm has not been evaluated in other studies, aside from the original study where it was first validated. It must be noted that the Gaussian algorithm was not developed in our population, but just validated, since this algorithm was constructed using previously published data from a large meta‐analysis. This might make this algorithm less likely to be overfitted to our population and, therefore, less likely to underperform when applied to a different population. Since first‐trimester PE screening and aspirin prescription has been implemented in most countries across Europe, prospective external validation of the Gaussian algorithm in untreated populations seems unlikely. Therefore, a reasonable indirect approach to assess the predictive performance of the Gaussian algorithm is to compare it with the FMF algorithm, which has been extensively validated in various large populations. Although our results cannot be considered an external validation of the Gaussian algorithm, the similar accuracies of both algorithms suggest that the FMF algorithm is unlikely to outperform the Gaussian algorithm in our population where it is being routinely used in most maternities since 2018. For this reason, we believe that the Gaussian algorithm might be a reasonable alternative to the FMF algorithm for those settings where the latter cannot be applied because of ultrasonographers performing UtAPI both transabdominally and transvaginally or for settings measuring biomarkers for the aneuploidy and PE screenings before 11 weeks. The results of this study are relevant since the Gaussian algorithm is already being implemented in other countries aside from Spain.

Additionally, as seen in previous studies,^23^ we confirm that PAPP‐A does not increase the predictive accuracy of any of the algorithms when PlGF was being used; however, when PlGF is not available, PAPP‐A could increase DR by 5% with some marker combinations. Finally, we observed that a single measurement of MAP could decrease the predictive accuracy of the FMF algorithm; therefore, the appropriate methodology (the average of two measurements in both arms simultaneously) should be performed when using this algorithm.

One of the main strengths of the study includes the prospective enrollment of patients. Furthermore, this study was performed within the context of routine clinical practice and patients were seen by their usual physicians, making the results more reliable and applicable in routine care settings. Moreover, this is the first study assessing the performance of the FMF algorithm exclusively in a Spanish cohort and in a clinical setting where MAP was measured once and only in one arm, showing similar results to those reported in the original study, for most combinations of markers. Despite a previous study showing that prediction of PE is similar when biomarkers are measured before or after 11 weeks,^6^ the FMF algorithm was designed with biomarkers assessed between 11 ± 0 and 13 ± 6 weeks. In this study, biomarkers were measured before 11 ± 0 weeks in 1675 (63.4%) women. Therefore, another remarkable strength of our work is that it provides evidence of the applicability of the FMF and Gaussian algorithms before and after 11 weeks for predicting PE and SGA.

The main limitation of our study is the low number of cases with early‐onset SGA and early‐onset PE and the relatively low number of cases with preterm SGA and preterm PE. Additionally, indication for elective delivery of SGA fetuses based on Doppler and cardiotocogram findings may be different when using other fetal growth restriction protocols. However, Doppler and cardiotocogram classification is uniform in Spain, where the Gaussian algorithm is widely used. Another limitation to be noted is that the technique for MAP measurements may potentially reduce the FMF algorithm's performance and could explain its lower AUC versus the Gaussian algorithm for some marker combinations.

## 5 CONCLUSIONS

This study shows that the first‐trimester Gaussian and FMF algorithms have similar predictive performances for PE and SGA in a Spanish population within a routine care setting. The accuracy of the FMF algorithm in our study was similar to that reported in previous studies, adding evidence to its external validity.

## AUTHOR CONTRIBUTIONS

Berta Serrano, MD; Erika Bonacina, MD; Pablo Garcia‐Manau, MD; Manel Mendoza, MD, PhD; and Elena Carreras, MD, PhD, had full access to all of the data in the study and take full responsibility for the integrity of the data and accuracy of the data analysis. Berta Serrano, MD; Erika Bonacina, MD; Pablo Garcia‐Manau, MD; Manel Mendoza, MD, PhD; and Elena Carreras, MD, PhD, conceived and designed the study. Berta Serrano, MD; Erika Bonacina, MD; Carlota Rodo, MD, PhD; Pablo Garcia‐Manau, MD; María Ángeles Sanchez‐Duran, MD, PhD; María Pancorbo, MD; Cristina Forcada, MD; María Teresa Murcia, MD; Ana Perestelo, MD; and Mireia Armengol‐Alsina, MD, contributed to literature research. Berta Serrano, MD; Erika Bonacina, MD; Carlota Rodo, MD, PhD; Pablo Garcia‐Manau, MD; María Ángeles Sanchez‐Duran, MD, PhD; María Pancorbo, MD; Cristina Forcada, MD; María Teresa Murcia, MD; Ana Perestelo, MD; and Mireia Armengol‐Alsina, MD, contributed to data collection and confirmation. Berta Serrano, MD; Erika Bonacina, MD; Pablo Garcia‐Manau, MD; and Manel Mendoza, MD, PhD, contributed to data analysis. Berta Serrano, MD; Erika Bonacina, MD; Pablo Garcia‐Manau, MD; Manel Mendoza, MD, PhD; and Elena Carreras, MD, PhD, contributed to data interpretation. Berta Serrano, MD, Erika Bonacina, MD; and Manel Mendoza, MD, PhD, were in charge of writing the article draft. All authors made substantial revisions to the article. All authors read and approved the final article.

## CONFLICTS OF INTEREST

Manel Mendoza, MD, PhD, received lecture fees by Roche diagnostics. The other authors report no conflicts of interest.

## Data Availability

The data that support the findings of this study are available from the corresponding author upon reasonable request.
